# Malignant Paroxysmal Supraventricular Tachycardia

**Published:** 2010-12-26

**Authors:** I Anguera, J Gonzalez-Costello, X Sabate

**Affiliations:** Division of Cardiology, Bellvitge Hospital, University of Barcelona, Barcelona, Spain

**Keywords:** atrial fibrillation, ablation, remote magnetic navigation, cryoballoon

## Case Report

**Figure 1:**
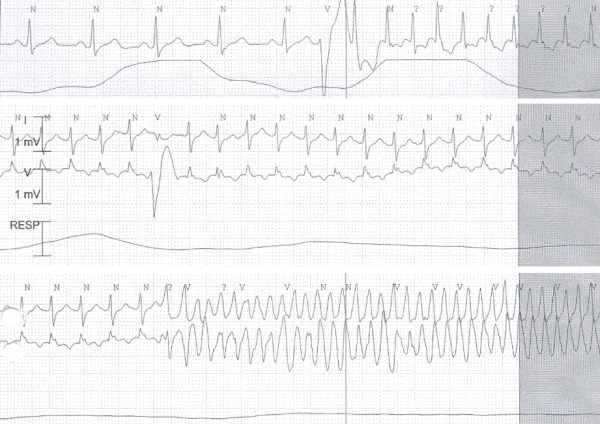
The upper pannel shows supraventricular tachycardia that was initiated by a ventricular premature beat. In the two lower pannels the supraventricular tachycardia degenerated to polymorphic ventricular tachycardia after 2 shortly coupled ventricular premature beats. The patient lost consciousness and had to be cardioverted.

A 63 year old male had a sudden episode of cardiac arrest during travel. He was resuscitated and cardioverted successfully with a semi-automatic defibrillator. The first ECG showed atrial fibrillation with inferior Q waves and he spontaneously reverted to sinus rhythm over the next few hours. The patient had been asymptomatic up to the episode of cardiac arrest and referred no prior history of ischemic heart disease. An acute ischemic event was ruled out because there was neither ST segment elevation on the surface electrocardiogram nor cardiac enzyme elevation. An echocardiogram showed localized inferior akinesia and preserved left ventricular ejection fraction. While being monitored over the next 2 days we documented recurrent episodes of supraventricular tachycardia (SVT) that converted to sinus rhythm after carotid sinus massage. On three occasions these episodes of SVT degenerated to syncopal polymorphic ventricular tachycardia (VT) that had to be cardioverted (Figure 1). The patient stopped having arrhythmias after infusion of amiodarone. An electrophysiological study demonstrated dual AV nodal physiology, and an atypical (slow-slow) atrioventricular nodal reentrant tachycardia (AVNRT) was induced. Radiofrequency catheter ablation of the slow pathway was successfully performed. After ablation, several episodes of non-sustained polymorphic VT were induced with a single extrastimulus. Coronary angiography showed chronic occlusion of the right coronary artery without intracoronary thrombus formation. Due to the absence of signs of ischemia or recent coronary occlusion, the patient was not revascularized. AVNRT was considered to be a trigger of malignant ventricular arrhythmias, but given the high risk of recurrence of sudden death despite successful ablation of the slow pathway a defibrillator (ICD) was implanted. After a follow-up of 9 months, the patient remains asymptomatic and has had no therapies delivered by the defibrillator.

## Discussion

This case illustrates the unusual case of a supraventricular tachycardia degenerating to VT in a patient with ischemic heart disease. Tachycardia-induced tachycardia is a rare condition. Cases have been described of AVNRT coincident with right ventricular outflow tract tachycardia or with left ventricular tachycardia [1]. In some cases where AVNRT spontaneously triggered VT, catheter ablation of the slow pathway did not suppress subsequent inducibility of VT and ablation of both substrates of arrhythmia is recommended [2].  Monomorphic VT is a frequent finding in patients with an old myocardial infarction, in which the scar represents the pathological substrate responsible for the re-entrant circuit. Polymorphic VT is a less frequent event in patients with chronic ischemic heart disease, both as a primary arrhythmia and as a finding during programmed ventricular stimulation, specially in patients after an episode of aborted sudden death [3-5].  Polymorphic VT is more related to additional factors like electrolyte imbalance, ischemia, long QT, Brugada syndrome, catecholaminergic polymorphic VT, but all these factors were excluded in our case [6]. The presence of ischemic heart disease with an old myocardial infarction without evidence of an acute ischemic milieu, recovered sudden death and inducibility of non-sustained polymorphic VT after successful slow pathway ablation lead us to implant an ICD to our patient.
